# A Qualitative Study of Emergency Department Delirium Prevention Initiatives

**DOI:** 10.56392/001c.55690

**Published:** 2022-12-06

**Authors:** Anita Chary, Shan W Liu, Ilianna Santangelo, Kyler M. Godwin, Christopher R. Carpenter, Aanand D Naik, Maura Kennedy

**Affiliations:** 1 Department of Medicine, Baylor College of Medicine; 2 Center for Innovations in Quality, Effectiveness and Safety, Michael E. DeBakey VA Medical Center; 3 Department of Emergency Medicine, Baylor College of Medicine; 4 Harvard Medical School; 5 Department of Emergency Medicine, Massachusetts General Hospital; 6 Department of Emergency Medicine, Barnes Jewish Hospital, Washington University School of Medicine, Emergency Care Research Core; 7 University of Texas School of Public Health, UT Health Science Center; 8 University of Texas Health Consortium on Aging

**Keywords:** delirium, prevention, geriatrics, emergency department

## Abstract

**Background:**

Delirium is a serious but preventable syndrome of acute brain failure. It affects 15% of patients presenting to emergency care and up to half of hospitalized patients. The emergency department (ED) often represents the entry point for hospital care for older adults and as such is an important site for delirium prevention.

**Objective:**

We sought to characterize delirium prevention initiatives in EDs in the United States and Canada.

**Methods:**

We conducted qualitative interviews with 16 ED administrators representing 14 EDs with delirium prevention initiatives. We used a combined deductive-inductive approach to code responses about involved staff, target patient population, and delirium prevention activities.

**Results:**

ED delirium prevention initiatives were largely driven by bedside nurses and occurred on an ad hoc basis, rather than systematically. Due to resource limitations, three EDs targeted older adults with high-risk conditions for delirium, rather than all patients age 65 and over. The most common delirium prevention interventions were offering assistive sensory devices (hearing amplifiers, reading glasses), having a toileting protocol, and offering patients food and drink.

**Conclusions:**

As minimal evidence exists about effective ED delirium prevention practices, low-cost and low-risk activities outlined by study participants are reasonable to use to improve patient experience and staff satisfaction.

## INTRODUCTION

Delirium is a syndrome of acute confusion and brain dysfunction that is common in older adults and is associated with high morbidity, mortality, and billions of dollars of annual healthcare costs.^1^ Delirium spans multiple healthcare settings, but it is a particularly important issue in the emergency department (ED).^2^ EDs often serve as an entry point into hospital care. ED environments also have numerous features that can contribute to the development of delirium, including crowding, hallway care, noise, and lack of windows.^3,4^ Indeed, a scoping review about ED delirium reports that delirium affects an estimated 6–38% of emergency department (ED) patients,^2^ and a recent meta-analysis reports an delirium prevalence of 15.2% in older adult ED patients.^5^ Importantly, delirium is even more common among hospitalized older adults, affecting up to 50% of hospitalized patients.^1^ Thirty to 40% of cases of delirium are preventable.^1^ Prevention of delirium in the ED can translate to numerous potential downstream health outcome improvements, including within the inpatient setting, and reduced healthcare spending.

Several delirium prevention strategies are suggested in the Geriatric Emergency Department guidelines, a set of recommendations for the ED care of older adults developed by consensus by leading emergency medicine and geriatrics organizations.^6^ The ADEPT tool, an educational resource developed by leaders in ED delirium research about strategies for prevention, assessment, and management, offers similar recommendations.^7^ These delirium prevention strategies include managing patients’ pain and other symptoms, providing sensory aids, ensuring hydration and nutrition, and encouraging mobility and caregiver visitation. A recent systematic review determined that melatonin and multi-factorial interventions are the most effective for prevention of ED delirium.^8^ However, while national guidelines and research studies exist, little is known about the current state of ED delirium prevention, specifically regarding real-world examples of how it occurs in practice.

This research sought to characterize delirium prevention initiatives for older adults in EDs in the United States and Canada. Understanding current practice in EDs that have adopted delirium prevention activities can inform planning and uptake of ED delirium prevention in other institutions.

## METHODS

We performed qualitative interviews with 16 administrators from 14 EDs with a delirium prevention initiative. We asked participants to describe their ED delirium prevention initiative, as part of a larger study about implementation of ED delirium initiatives. Interview questions are available as supplementary material. The larger study included 23 administrators leading an ED delirium initiative who represented 20 EDs. Interviews were conducted from December 2021 to June 2022 with emergency nurses, physicians, and allied health professionals.

We recruited participants by email in two ways. First, we sent invitations to the list-servs of the American College of Emergency Physicians Geriatric Emergency Department Collaborative and the Society for Academic Emergency Medicine’s Academy of Geriatric Emergency Medicine. Second, we contacted individuals known to lead ED delirium initiatives based on their academic publications, national lectures, and social media posts.

Interviews were conducted using an online audio-visual platform by a non-clinician research assistant (IS) with prior qualitative interview experience and supervised by a qualitative methods expert with a PhD in Anthropology (ANC). The RA had no prior relationships with study participants. Twelve one-on-one interviews were performed and two interviews were conducted with two interviewees representing one institution. Interviews lasted 15 to 50 minutes, excluding the informed consent procedure. Interview length varied as the larger research study asked participants about delirium prevention, detection, and management programs; interviews were of longer duration in sites with all three types of initiatives. On average, interviews lasted 36 minutes. Participants did not receive compensation. Audio recordings of interviews were professionally transcribed and reviewed for accuracy by the study team. This study did not involve generating field notes from interviews or returning transcripts to participants for comments.

**Figure 1.**
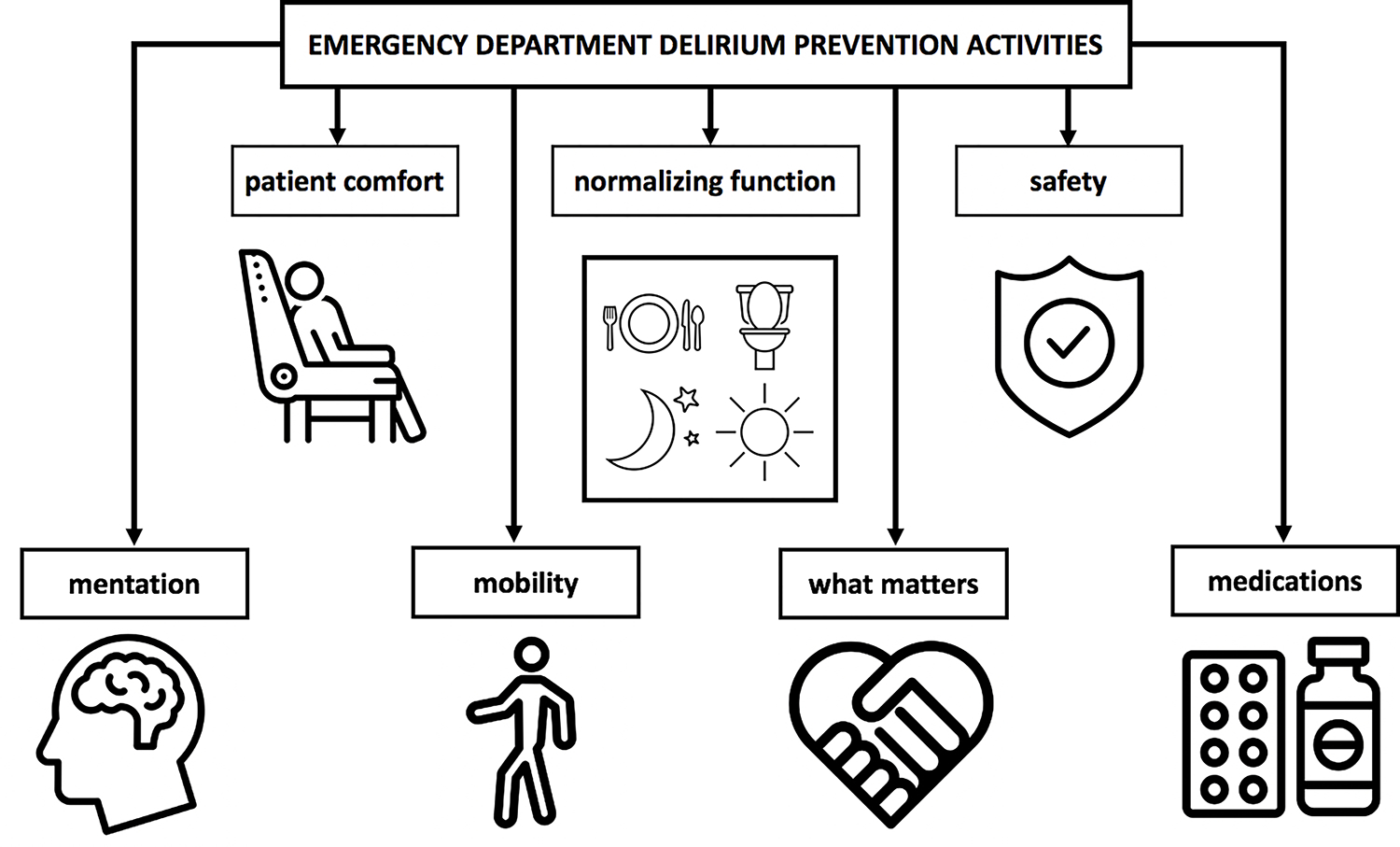
Coding Tree for ED Delirium Prevention Activities

This research used a methodological orientation of conceptual content analysis, which seeks to determine the existence of concepts and quantify them in text.^9^ Two study team members (ANC, IS) used a deductive approach to categorize responses based on: involved staff, target population, and types of delirium prevention activities. Coded data were then iteratively reviewed by all study team members for common themes.^10^ Types of delirium prevention activities were categorized into themes using a combined deductive-inductive approach. From initial review of transcripts, the team noted that many responses fell under categories of the 4Ms of an Age-Friendly Health System framework, which prioritizes what matters most (understanding and aligning care with a patient’s health goals and preferences), mobility (older adults moving safely and maintaining functional status), mentation (addressing delirium, dementia, and depression), and medications (using age-friendly medications that do not impede mental and physical functional status).^11^ For delirium prevention activities that did not fit under these categories, researchers held consensus discussions to classify responses related to patient comfort, normalizing function, and safety. The coding tree for delirium prevention activities is depicted in Figure 1. Researchers used Microsoft Excel to facilitate analysis. Data saturation was achieved by 10 interviews; no new themes subsequently emerged.

The Institutional Review Boards of Baylor College of Medicine, Houston, TX (H-50838) and Partners Healthcare, Boston, MA (2021P001558) approved this research. We report methods using the Consolidated Criteria for Reporting Qualitative Research (COREQ).^12^

## RESULTS

Sixteen interviewees representing 14 EDs with an ED delirium prevention initiative participated in the study. Interviewees included 9 physicians, 3 nurses, 3 advanced practice clinicians, and 1 allied health professional. Because the invitation to participate in the interview was distributed to list-servs comprised of hundreds of individuals, we are unable to report a response rate. The EDs interviewees represented were located in the United States (n=14) and Canada (n=2). Over half (n=8) were accredited as geriatric emergency departments by the American College of Emergency Physicians.^13^ About half of EDs had used the Geriatric ED Guidelines^6^ (n=8, 57%) and/or an ED delirium educational resource called ADEPT^14^ (n=6, 43%) to inform their initiative. Further sample characteristics are detailed in Table 1. Three major themes emerged regarding common preventive interventions, the informal nature of delirium prevention initiatives, and approaches to the target population for intervention. Data saturation with these themes was achieved by ten interviews.

### Common ED delirium prevention interventions.

Interviewees reported their EDs employed numerous interventions to prevent delirium (Table 2). The most common delirium prevention interventions were related to mentation and normalizing function: offering assistive sensory devices (hearing amplifiers, reading glasses); having a toileting protocol (ensuring a patient toileted at least once during each shift, communicating within the care team about assistance needed to toilet and last time a patient toileted); and offering patients food and drink. About one third of sites (n=5) employed a geriatrics cart containing sensory devices and comfort items or had available geriatrics activity kits, which either represented part or the entirety of their delirium prevention initiative.

### Informal nature of ED delirium prevention interventions.

Overall, interviewees described that they had created resources and pathways to prevent delirium in their EDs, but did not consider their ED delirium prevention formal, protocolized, or systematically incorporated into patient care. Rather, EDs left use of delirium prevention resources and measures to the discretion of patients’ care teams. All interviewees reported that their delirium prevention activities were largely initiated and carried out by bedside nurses. Some institutions also involved ancillary staff including technicians, social workers, and personal support workers. Interviewees perceived that physicians and advanced practice clinicians had a lesser role in initiating delirium prevention activities.

### Target population for delirium prevention.

EDs took variable approaches to which patients they targeted for delirium prevention. Most sites did not formally define a target population for delirium prevention, but rather left initiation of prevention activities to the discretion of the care team, and predominantly to bedside nurses. A few interviewees expressed that in their practice setting, it would not be feasible to direct delirium prevention efforts towards all patients aged 65 and older, as recommended in the geriatric ED guidelines. These EDs targeted boarding patients (n=1, 7%), patients ages 75+ (n=1, 7%), or patients over 65 with specific chief complaints or diagnoses deemed high-risk for developing delirium, such as falls, hip fractures, or sepsis (n=1, 7%).

## DISCUSSION

This study highlights common practices in ED delirium prevention, a reliance on bedside nurses to initiate delirium prevention, and flexible approaches that target high-risk patients for prevention activities. Overall, within our sample, delirium prevention activities were not protocolized or conducted systematically. These findings have implications for routine emergency care of older adults.

Delirium is receiving increasing attention as an important problem in emergency medicine.^2,15^ A recent study found that 22% of EDs that received geriatric accreditation from the American College of Emergency Physicians had implemented delirium screening protocols.^16^ Our research highlights that ED delirium prevention efforts in emergency settings are under development and likely underutilized.

In the ED setting, there is limited evidence to support specific delirium prevention initiatives.^2,8^ This contrasts with inpatient settings where delirium prevention is well-researched, and for which evidence exists about interventions that do and do not confer benefits.^1,17–19^ The absence of robust, evidence-based research to support large-scale ED delirium prevention favors implementation of low to no-risk activities that impact patient experience and staff satisfaction. Our interviews identified several options that can be adopted at low cost (e.g. sensory aids) or no cost (e.g. toileting protocol). Other literature demonstrates that geriatric assist devices and comfort carts help patients more fully engage in ED care, facilitate communication, and improve patient and staff experience.^20–22^

In our sample, ED delirium prevention efforts were implemented at the discretion of bedside nurses. This contrasts sharply with the inpatient approach to delirium prevention. For example, the Hospital Elder Life Program (HELP) is a widely used multi-component intervention that prevents delirium and functional decline in hospitalized older adults.^18^ HELP uses a set of highly-structured staff education and care interventions that are systematically assigned, performed, and tracked by interdisciplinary teams.^17^ Core interventions (e.g. orientation, sleep enhancement, mobilization) are performed by a variety of care team members, including nurses, geriatricians, pharmacists, and volunteers, are rigorously tracked through interdisciplinary rounds and promoted through ongoing educational curricula.^18^ The ad hoc approach to ED delirium prevention outlined by our interviewees arises from resource limitations and a lacking evidence base for specific ED interventions. The informality with which ED delirium prevention activities occur may preclude research into their effectiveness. Research about effective ED delirium prevention interventions will require more formalized and systematic programs than those described herein.

Preferentially engaging emergency nurses in delirium prevention is pragmatic, given the amount of time emergency nurses spend at bedside compared to other staff members. Additionally, many of the interventions described herein fall under the purview of routine nursing activities. Literature suggests that emergency nurses recognize a need for education about delirium and appreciate their role in improving delirium-related patient experience and outcomes.^23,24^ However, any ED delirium prevention initiatives must take into account bedside nurses’ already extensive patient care responsibilities and attempt to minimize additional workload.^2^ There may be a potential role for greater involvement of frontline clinicians in initiating discussions with other care team members and specialists about delirium prevention.

Finally, our interviewees described that it was not feasible for EDs to target all older adults for delirium prevention based on resource limitations. Geriatric ED guidelines that outline best practices for the emergency care of older adults—including delirium prevention—are aspirational and may be difficult to implement in routine care.^25^ Taking an incremental approach that targets high-risk patients for ED delirium prevention—e.g. those with falls, hip fractures, sepsis, or those boarding and awaiting admission—is a reasonable strategy that could be used in other institutions with limited resources. Evidence suggests that older adults receiving care in ED hallways or those with an ED length of stay is >10 hours are also high risk for developing delirium.^3,4^ Prioritizing these patients for delirium prevention interventions is particularly important given the current prevalence of ED boarding and crowding related to the COVID-19 pandemic.^26^ Hospital throughput changes to mitigate ED crowding could help meaningfully address ED delirium prevention at the systems level, but have historically been difficult to enact,^26^ making the activities identified in this research all the more important.

## LIMITATIONS

This study faces several limitations. Over half of interviewees in this investigation were from geriatric-accredited institutions, whose unique commitment to and resources for geriatric emergency medicine do not reflect routine emergency medicine practice settings. However, interviews did identify several practical, low- or no-cost measures that could be adopted at sites without geriatric-specific resources. We interviewed administrators and geriatrics champions familiar with their institution’s ED delirium prevention initiative. This study does not provide information about how and when frontline emergency staff or clinicians decide to use delirium prevention resources, an important area for future study. While thematic saturation was achieved in this study, a relatively small number of EDs was represented. While we provide quantitative data to demonstrate the most common delirium prevention activities used in this sampled population, these may not reflect commonly adopted activities in other EDs with delirium prevention initiatives. Additionally, this study relied on reports from one or two individuals affiliated with each institution, and recall bias may have affected the reported delirium prevention activities. Thus, our findings about delirium prevention activities are likely not comprehensive. Despite these limitations, our study has notable strengths including its rigorous methods, achievement of thematic saturation, and characterization of current practice among early adopters of ED delirium prevention.

## Supplementary Material

Supplement - Interview Guide

## Figures and Tables

**Table 1.** Characteristics of Interviewees and EDs Represented with Delirium Prevention Initiative

Characteristic	N (%)

Interviewee’s professional role	
Physician	9 (56%)
Nurse	3 (19%)
Advanced practice clinician	3 (19%)
Allied health professional	1 (6%)

Country of Practice	
United States	12 (86%)
Canada	2 (14%)

Language of care delivery	
English	12 (86%)
English and French	2 (14%)

Teaching Institution	14 (100%)

Geographic Setting	
Urban	10 (71%)
Suburban	4 (29%)

ED geriatric accreditation status	
Accredited	8 (57%)
Non-accredited	6 (43%)

Resources used to inform initiative	
Geriatric ED Guidelines	8 (57%)
ADEPT	6 (43%)
Initiative predated publication of above resources	1 (7%)

The first row details professional roles of N=16 interviewees who represent 14 EDs. All subsequent rows detail attributes of N=14 EDs.

**Table 2.** ED Delirium Prevention Strategies

Category	Strategy	N* (%)

Mentation	Provide hearing amplifier, hearing aid batteries	8 (57%)
	Provide reading glasses, glasses repair kit	7 (50%)
	Cognitive games (crossword puzzle, reading materials, playing cards, word search, coloring tools, crayons/coloring pencils)	5 (36%)
	Clock/calendar in room	2 (14%)
	Instruct family about delirium and remind them to bring hearing aid, reading glasses	1 (7%)
	Allow uninterrupted sleep	1 (7%)

Mobility	Assess mobility and mobilize patient during each shift	4 (29%)
	Designate technician as “ambulator” to walk with geriatric patients	1 (7%)
	Chair at bedside	1 (7%)
	Remove tethers when possible (Foley, IV tubing, monitor cables)	1 (7%)

Medication	Avoid high-risk medications (benzodiazepines, opioids, anticholingergic, antipsychotics)	2 (14%)
	Perform medication reconciliation	1 (7%)

Matters Most	Encourage visitation	4 (29%)
	Have volunteer at bedside for company	1 (7%)

Comfort	Warming items (blanket, hat, mittens)	2 (14%)
	Move boarding observation/admitted patients to low stimulation environment (single-person room with doors)	2 (14%)
	Ear plugs	2 (14%)
	Aromatherapy	1 (7%)
	Lighting	1 (7%)
	Music	1 (7%)
	Face/sleep mask	1 (7%)

Normalizing Function	Ensure hydration	8 (57%)
	Provide regular meals	8 (57%)
	Toileting protocol and care team communication regarding toileting status and assistance needed	6 (43%)
	Bathing, brushing teeth	1 (7%)

Safety	Distraction tools to mitigate agitation	4 (29%)
	Move patient closer to nurses’ station for monitoring	2 (14%)
	Sitter at bedside for fall prevention	1 (7%)

* Data are reported by N=14 EDs represented by interviewees.
