# Supplementary material for: A Qualitative Study of Emergency Department Delirium Prevention Initiatives
Source: Delirium Commun. Author manuscript; Available in PMC 2024 Jul 19. (PMC11259403; doi:10.56392/001c.55690)
Supplement: Supplement - Interview Guide [file NIHMS1954659-supplement-Supplement_-_Interview_Guide.pdf]

Supplementary Material. Interview Questions about ED Delirium Prevention Initiatives

Question	*Prompts*
Please tell me about your delirium prevention initiative.	What actions does your institution take to prevent delirium?
How do you determine who receives the intervention?	What risk factors are used to identify someone at risk for delirium?
Who is responsible for initiating the delirium prevention program?	Triage Nurses? Primary/Bedside Nurses? Physician? Advanced Practice Provider?
